# Supplementary material for: Transient remodeling of gut metabolism supports juvenile growth and adult fitness in Drosophila
Source: Nat Commun. 2026 Apr 13;17:3458. doi: 10.1038/s41467-026-71776-3 (PMC13077097; doi:10.1038/s41467-026-71776-3)
Supplement: Supplementary file 1 — Supplementary Information [file 41467_2026_71776_MOESM1_ESM.pdf]

Supplementary Information for

Transient remodeling of gut metabolism supports juvenile growth and adult fitness in *Drosophila*

Lefranc *et al.*

*Corresponding author: Gilles Storelli (gilles.storelli@cos.uni-heidelberg.de)

This PDF file includes:

Supplementary Figures 1-7

Supplementary Tables 1-2

Supplementary References

a

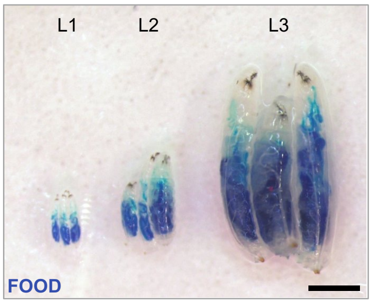

Supplementary Figure 1. Larvae feed continuously throughout development

(**a**) Representative pictures of larvae that have been fed food stained with blue dye. The stained food can be seen in the digestive system of L1, L2 and L3 larvae. Representative pictures of n=60 larvae from two independent experiments. Scale bar = 1 mm. Source data are provided as a Source Data file.

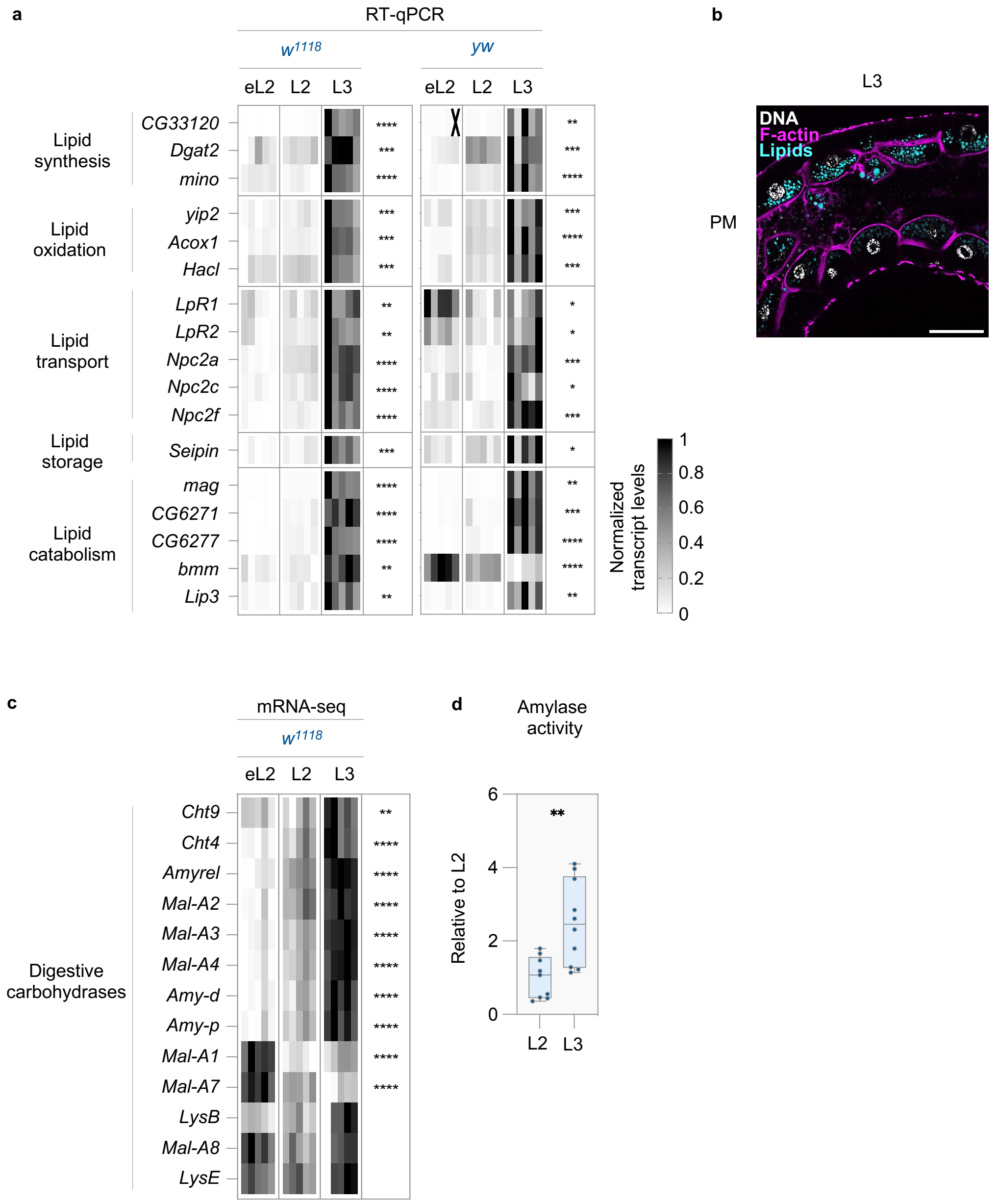
a
RT-qPCR
w1118
yw
eL2
L2
L3
eL2
L2
L3
Lipid synthesis
CG33120
Dgat2
mino
Lipid oxidation
yip2
Acox1
Hacl
Lipid transport
LpR1
LpR2
Npc2a
Npc2c
Npc2f
Lipid storage
Seipin
Lipid catabolism
mag
CG6271
CG6277
bmm
Lip3
Normalized transcript levels
1
0.8
0.6
0.4
0.2
0
b
L3
PM
DNA
F-actin
Lipids
c
mRNA-seq
w1118
eL2
L2
L3
Digestive carbohydrases
Cht9
Cht4
Amyrel
Mal-A2
Mal-A3
Mal-A4
Amy-d
Amy-p
Mal-A1
Mal-A7
LysB
Mal-A8
LysE
d
Amylase activity
Relative to L2
6
4
2
0
L2
L3

Supplementary Figure 2. Intestinal function is increased in L3 larvae

(**a-d**) Early L2 (eL2), late L2 (L2) and early L3 (L3) larvae were analyzed. (**a**) Levels of transcripts involved in lipid metabolism in w^{1118} and *yw* larval midguts. Gene groups are annotated with their main function, the term "Lipid catabolism" regroups digestive and intracellular lipases. Crosses in the heatmap indicate the biological replicates for which a given transcript was not detected. n=5 biological replicates. One representative experiment out of three is shown. (**b**) Cell nuclei, actin filaments (F-actin) and lipid droplets were stained with DAPI, phalloidin and BODIPY, respectively, in the posterior midgut (PM) of L3 larvae. Scale bar = 50 µm. Representative image from three independent experiments (n=15 biological replicates). (**c**) Levels of transcripts encoding digestive carbohydrases in w^{1118} larval midguts. n=5 biological replicates. (**d**) Amylolytic activity in the midguts of L2 and L3 larvae, normalized to protein content and presented relative to L2 controls. n=9 (L2) and 10 (L3) biological replicates from two independent experiments (see the methods for details about the contents of the biological replicates used in these assays). (**a and c**) Heatmaps depict transcript levels and statistically significant difference across timepoints. Transcript levels are represented using a greyscale (minimal and maximal values are represented by white and black, respectively, see methods for details). (**d**) Dots represent individual biological replicates, boxplots extend from the 25th to 75th percentile, whiskers extend from minimum to maximum, median is depicted as a line. (**a, c and d**) Asterisks indicate statistically significant differences between timepoints ((**a**) Kruskal-Wallis test, (**c**) 1-way ANOVA or (**d**) two-sided Student's t test). *$p \leq 0.05$, **$p \leq 0.01$, ***$p \leq 0.001$, ****$p \leq 0.0001$. Exact p-values are indicated in the Source data file.

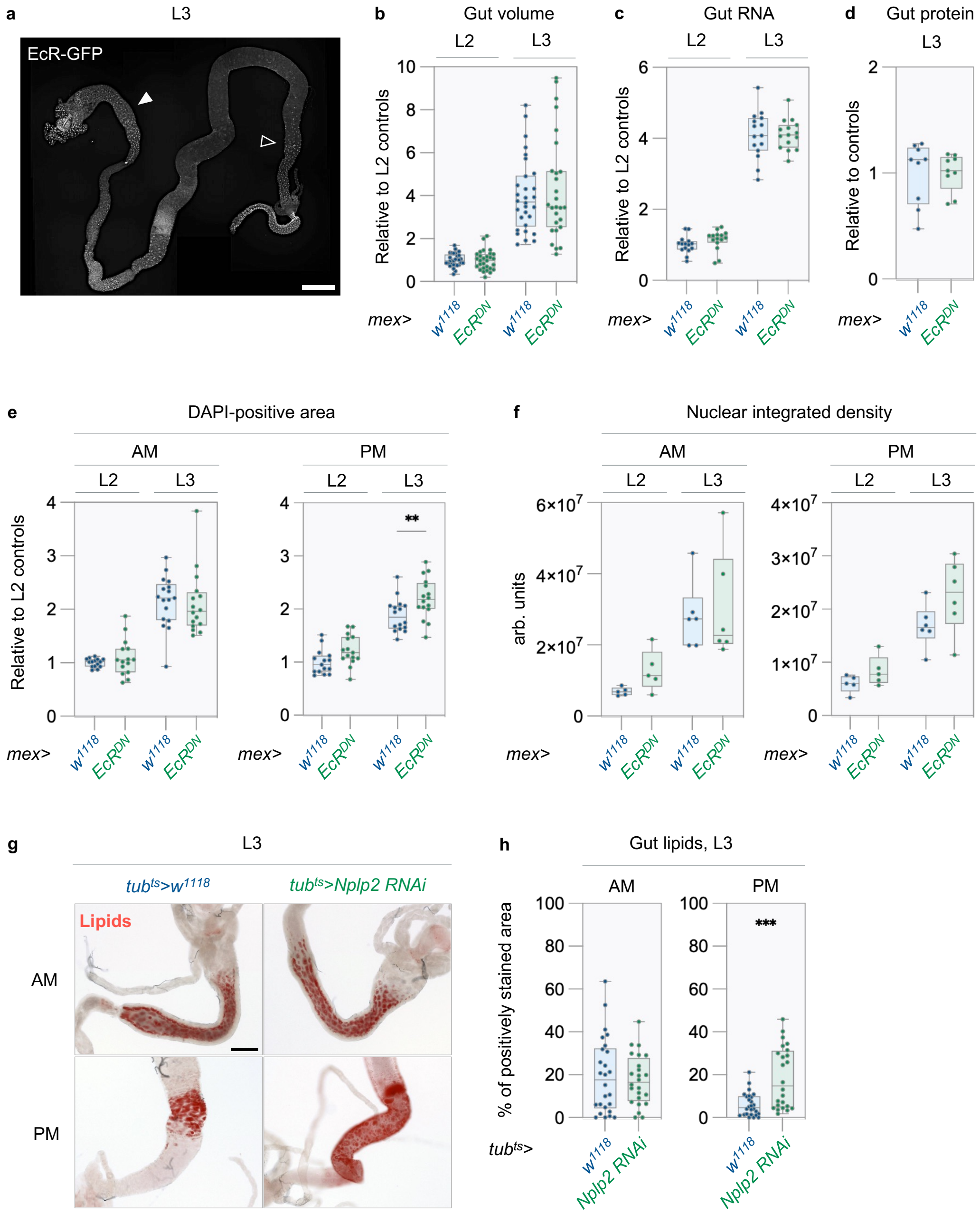

a
L3
EcR-GFP
b
Gut volume
L2
L3
Relative to L2 controls
mex>
w1118
EcRDN
c
Gut RNA
d
Gut protein
Relative to controls
e
DAPI-positive area
AM
PM
f
Nuclear integrated density
arb. units
g
tubts>w1118
tubts>Nplp2 RNAi
Lipids
h
Gut lipids, L3
% of positively stained area
tubts>
Nplp2 RNAi

Supplementary Figure 3. Suppression of EcR signaling does not reduce gut growth

(**a**) Anti-GFP antibody stains were used to visualize EcR in the midgut of *EcR-GFP* animals. The anterior and posterior midguts are indicated with closed and open arrowheads, respectively. Scale bar = 500 µm. Representative image from two independent experiments (n=20 biological replicates). (**b-f**) A dominant negative form of EcR (EcR^{DN}) was expressed in ECs with the *mex-GAL4* driver (*mex>*EcR^{DN}). *mex>*w^{1118} animals were used as controls (**b**) Midgut volume, relative to L2 controls. mex>w^{1118}: n=29 (L2) and 30 (L3). *mex>*EcR^{DN} : n=29 (L2) and 27 (L3). Biological replicates from three independent experiments. (**c**) RNA levels in midguts, relative to L2 controls. n=15 biological replicates from four independent experiments. (**d**) Protein levels in midguts, relative to controls. n=9 biological replicates from three independent experiments. (**e**) Nuclear size estimated from DAPI stains in the anterior midgut (AM) and posterior midgut (PM), relative to L2 controls. mex>w^{1118}: n=15 (L2), 17 (AM L3) and 16 (PM L3). *mex>*EcR^{DN}: n=15 (L2) and16 (L3) from three independent experiments. (**f**) Intensity of DAPI staining as an estimate of ploidy, relative to L2 controls. n=5 (L2) and n=6 (L3) midguts. One representative experiment out of three is show. (**g and h**) Lipids were stained with ORO in the AM and PM of L3 larvae with systemic silencing of the lipoprotein Nplp2 after hatching (tub^{ts}*>Nplp2 RNA*$_i$). tub^{ts}>w^{1118} were used as controls, see methods for details. (**g**) Representative images, scale bar = 200 µm. (**h**) quantification of ORO-stained area as a percentage of total area. (**g and h**) n=24 (tub^{ts}>w^{1118}) and 26 (tub^{ts}*>Nplp2 RNA*$_i$) midguts from three independent experiments. (**b-f, h**) Dots represent individual biological replicates, boxplots extend from the 25th to 75th percentile, whiskers from minimum to maximum, median is depicted as a line. Asterisks indicate statistically significant differences (**b, c, e and f**) between genotypes at a given timepoint (2-way ANOVA with Šidak's multiple comparisons) and (**d, h**) between genotypes ((**d**) two-sided Student's t test and (**h**) two-sided Mann-Whitney test). (**b**) p=0.9996 (L2) and 0.6469 (L3); (**c**) p= 0.6463 (L2) and 0.992 (L3); (**d**) p=0.9725; (**e**) p=0.7847 (AM L2), 0.9453 (AM L3), 0.0943 (PM L2), 0.0094 (PM L3); (**f**) p= 0.585 (AM L2), 0.9178 (AM L3), 0.6469 (PM L2), 0.0791 (PM L3); (**h**) p=0.9728 (AM) and 0.0006 (PM). *p≤0.05, **p≤0.01, ***p≤0.001. Source data are provided as a Source Data file.

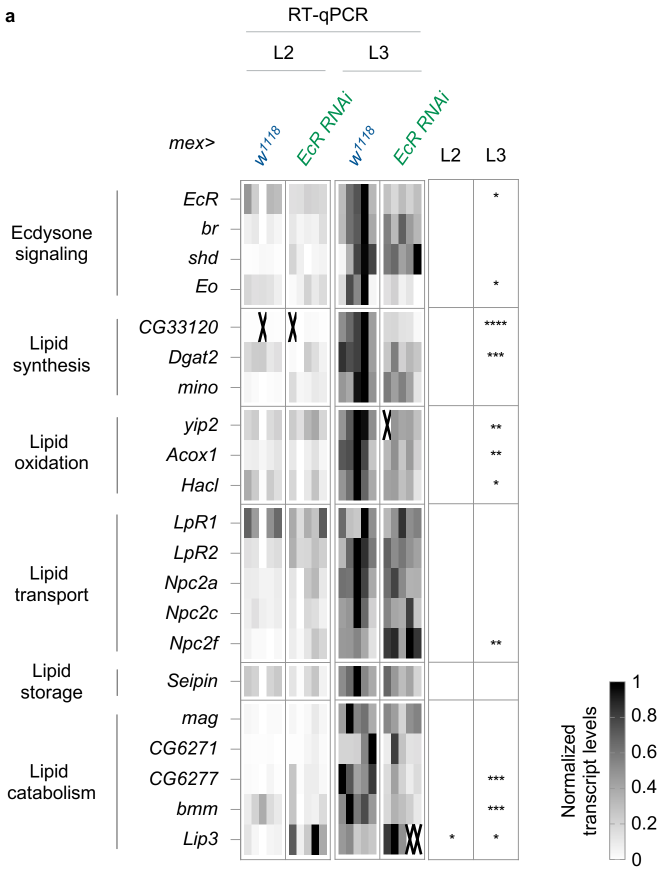

a
RT-qPCR
L2
L3
mex>
w1118
EcR RNAi
w1118
EcR RNAi
L2
L3
Ecdysone signaling
EcR
br
shd
Eo
Lipid synthesis
CG33120
Dgat2
mino
Lipid oxidation
yip2
Acox1
Hacl
Lipid transport
LpR1
LpR2
Npc2a
Npc2c
Npc2f
Lipid storage
Seipin
Lipid catabolism
mag
CG6271
CG6277
bmm
Lip3
Normalized transcript levels
1
0.8
0.6
0.4
0.2
0

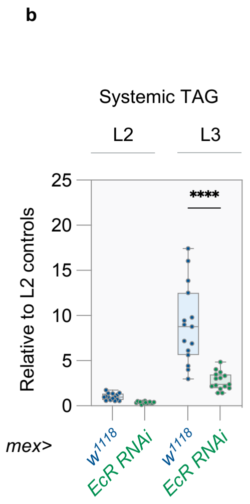

b
Systemic TAG
L2
L3
Relative to L2 controls
25
20
15
10
5
0
****
mex>
w1118
EcR RNAi
w1118
EcR RNAi

Supplementary Figure 4. EcR signaling regulates intestinal lipid metabolism

(**a and b**) Ecdysone signaling was suppressed in ECs by expressing *UAS-EcR RNAi* with the *mex-GAL4* driver (*mex>EcR RNAi*). Controls are progenies of *mex-GAL4* females and w^{1118} males (*mex>*w^{1118}). (**a**) Levels of transcripts involved in ecdysone signaling and lipid metabolism were scored with RT-qPCR in L2 and L3 midguts. Gene groups are annotated with their main function. "Lipid catabolism" includes digestive and intracellular lipases. The heatmap depicts transcript levels and statistically significant differences between genotypes at a given timepoint. Transcript levels are represented using a greyscale (minimal and maximal values are represented by white and black, respectively, see methods for details). Crosses indicate the biological replicates for which a given transcript was not detected. n=5 biological replicates, each containing several midguts, per genotype and timepoint (see the methods for details about the contents of the biological replicates used in these assays). One representative experiment out of three is shown. (**b**) Total triglyceride content in L2 and L3 larvae, presented relative to L2 controls. n=15 biological replicates, each containing several larvae, per genotype and timepoint from three independent experiments (see the methods for details about the contents of the biological replicates used in these assays). Dots represent individual biological replicates, boxplots extend from the 25th to 75th percentile, whiskers extend from minimum to maximum, median is depicted as a line. (**a and b**) Asterisks indicate statistically significant differences between genotypes at a given developmental stage (2-way ANOVA with Šidak's multiple comparisons). (**a**) Exact p-values are indicated in the Source data file. (**b**) $p=0.6945$ (L2) and $p<0.0001$ (L3). *$p\leq0.05$, **$p\leq0.01$, ***$p\leq0.001$, ****$p\leq0.0001$. Source data are provided as a Source Data file.

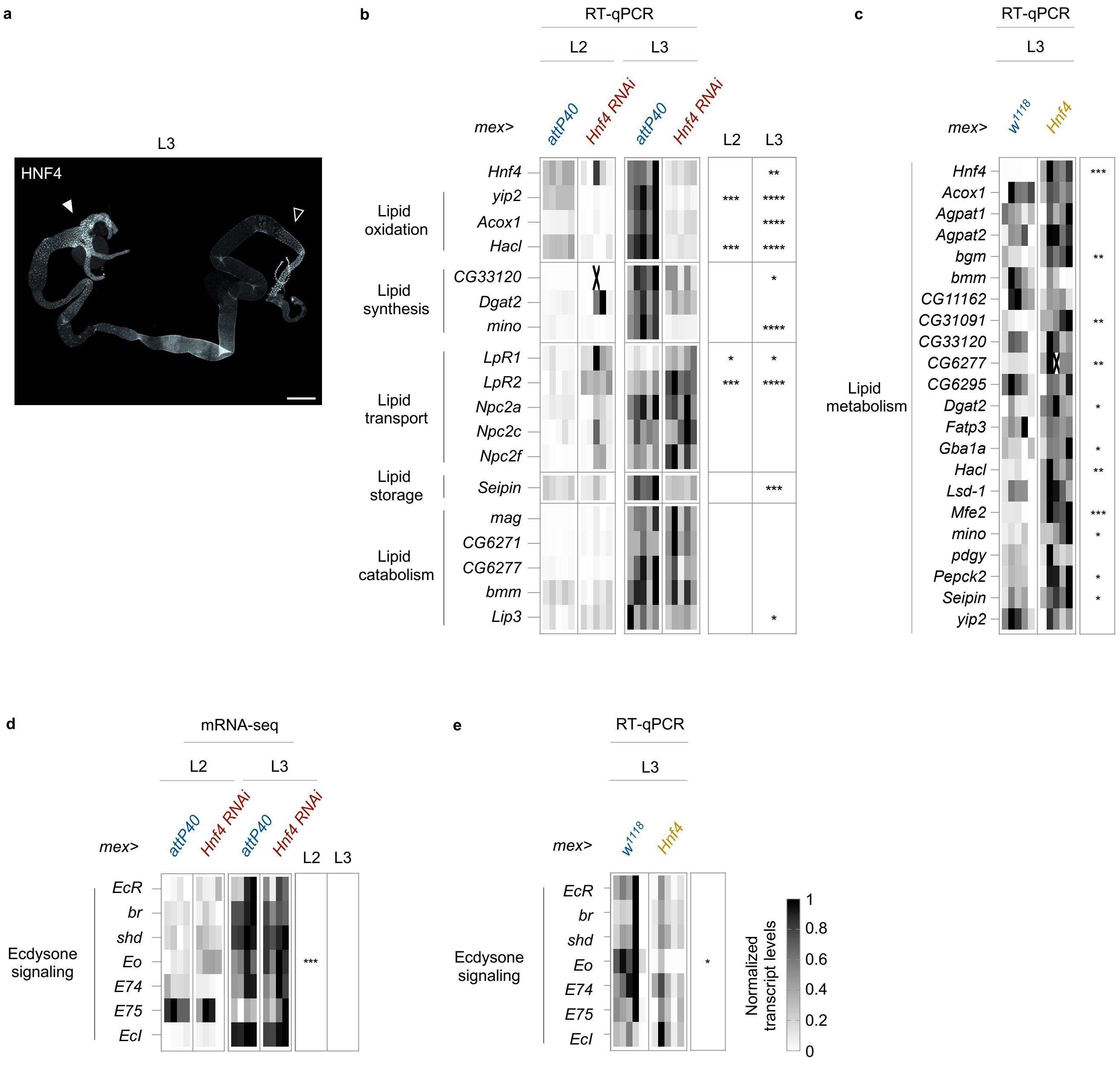

a
L3
HNF4
b
RT-qPCR
L2
L3
mex>
attP40
Hnf4 RNAi
attP40
Hnf4 RNAi
L2
L3
Hnf4
Lipid oxidation
yip2
Acox1
Hacl
Lipid synthesis
CG33120
Dgat2
mino
Lipid transport
LpR1
LpR2
Npc2a
Npc2c
Npc2f
Lipid storage
Seipin
Lipid catabolism
mag
CG6271
CG6277
bmm
Lip3
c
RT-qPCR
L3
mex>
w1118
Hnf4
Lipid metabolism
Hnf4
Acox1
Agpat1
Agpat2
bgm
bmm
CG11162
CG31091
CG33120
CG6277
CG6295
Dgat2
Fatp3
Gba1a
Hacl
Lsd-1
Mfe2
mino
pdgy
Pepck2
Seipin
yip2
d
mRNA-seq
L2
L3
mex>
attP40
Hnf4 RNAi
attP40
Hnf4 RNAi
L2
L3
Ecdysone signaling
EcR
br
shd
Eo
E74
E75
Ecl
e
RT-qPCR
L3
mex>
w1118
Hnf4
Ecdysone signaling
EcR
br
shd
Eo
E74
E75
Ecl
Normalized transcript levels
1
0.8
0.6
0.4
0.2
0

Supplementary Figure 5. HNF4 regulates the expression of genes involved in lipid metabolism in the midgut

(**a**) Antibody stains were used to visualize HNF4 in the anterior and posterior midgut (closed and open arrowheads, respectively) of L3 controls (*mex*>w^{1118}). Representative image from n=30 biological replicates from three independent experiments. Scale bar = 500 µm. (**b and d**) *Hnf4* was silenced in ECs by driving the expression of *UAS-Hnf4 RNAi* with the *mex-GAL4* driver (*mex>Hnf4 RNAi*). Controls are progenies of *mex-GAL4* females and males carrying the *attP40* site (*mex>attP40*). The levels of transcripts related to **(b)** lipid metabolism (RT-qPCR) or **(d)** ecdysone signaling (mRNA-seq) were scored in L2 and L3 midguts. **(b)** n=5 and **(d)** n=4 biological replicates, each containing several midguts (see the methods for details about the contents of the biological replicates used in these assays). For **(b)**, one representative experiment out of three is shown. (**c and e**) *Hnf4* was overexpressed in otherwise wild-type ECs by driving the expression of *UAS-Hnf4* transgene with the *mex-GAL4* driver (*mex>Hnf4*). Controls are progenies of *mex-GAL4* females and w^{1118} males (*mex*>w^{1118}). The levels of transcripts related to **(c)** lipid metabolism or **(e)** ecdysone signaling were scored with RT-qPCR in L3 midguts following *Hnf4* overexpression. (**c and e**) n=5 biological replicates, each containing several midguts (see the methods for details about the contents of the biological replicates used in these assays). One representative experiment out of three is shown. (**b-e**) Heatmaps depict transcript levels and statistically significant differences between genotypes at a given timepoint. Transcript levels are represented using a greyscale (minimal and maximal values are represented by white and black, respectively, see methods for details). Crosses in the heatmap indicate biological replicates for which a given transcript was not detected. Asterisks indicate statistically significant differences (**b and d**) between genotypes at a given timepoint (2-way ANOVA with Šidak's multiple comparisons) or (**c and e**) between genotypes (two-sided Mann Whitney or two-sided Student's t test based on the distribution of values). Exact p-values are indicated in the Source data file. *p≤0.05, **p≤0.01, ***p≤0.001, ****p≤0.0001. Source data are provided as a Source Data file.

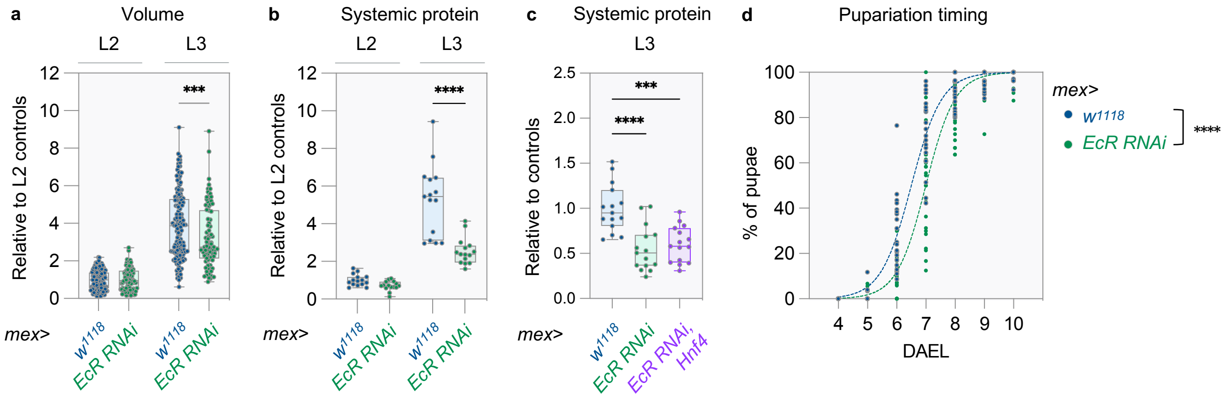
a
Volume
L2
L3
Relative to L2 controls
***
mex>
w1118
EcR RNAi
b
Systemic protein
L2
L3
Relative to L2 controls
****
mex>
w1118
EcR RNAi
c
Systemic protein
L3
Relative to controls
***
****
mex>
w1118
EcR RNAi
EcR RNAi, Hnf4
d
Pupariation timing
% of pupae
DAEL
mex>
w1118
EcR RNAi
****

Supplementary Figure 6. EcR acts in EC to support systemic growth and maturation

(**a-d**) Ecdysone signaling was suppressed in ECs by expressing *UAS-EcR RNAi*, alone or in combination with *UAS-Hnf4,* with the *mex-GAL4* driver (*mex>EcR RNAi* and *mex>EcR RNAi, Hnf4,* respectively). Controls are the progenies of *mex-GAL4* females and w^{1118} males (*mex*>w^{1118}). (**a**) Body volume, relative to L2 controls. n=126 (L2 *mex*>w^{1118}), 89 (L2 *mex>EcR RNAi*), 140 (L3 *mex*>w^{1118}) and 82 (L3 *mex>EcR RNAi*) animals, from two independent experiments. (**b and c**) Protein content was scored in (**b**) L2 and L3 larvae with inhibition of ecdysone signaling in ECs, and (**c**) L3 larvae in which *UAS-EcR RNAi* is expressed simultaneously with *UAS-Hnf4* in ECs. The data is presented relative to (**b**) L2 or (**c**) L3 controls. n=15 biological replicates, each containing several animals, per genotype and timepoint from three independent experiments (see the methods for details about the contents of the biological replicates used in these assays). (**d**) Pupariation was scored between D4-D10AEL following inhibition of ecdysone signaling in ECs. Values are plotted normalized to the total number of pupae. n=20 (*mex*>w^{1118}) and 27 (*mex>EcR RNAi*) biological replicates, each containing several animals, from three independent experiments (see the methods for details). The sigmoid curve represents non-linear least square regression fit of the data. Median time to pupariation extrapolated from the non-linear fit analysis: *mex*>w^{1118}=6.50 days, *mex*> *EcR RNAi* =7.00 days. (**a-d**) The dots represent individual biological replicates. (**a-c**) The boxplots extend from the 25th to 75th percentile, whiskers extend from minimum to maximum, median is depicted as a line. (**a-d**) Asterisks indicate statistically significant differences (**a and b**) between genotypes at a given timepoint (2-way ANOVA with Šidak's multiple comparisons) and (**c and d**) between genotypes ((c) 1-way ANOVA with Šidak's multiple comparisons, (**d**) extra sum-of-squares F test). (**a**) p=0.9906 (L2) and 0.0008 (L3); (**b**) p=0.6878 (L2) and p<0.0001 (L3); (**c**) p<0.0001 (*mex*>w^{1118} vs. *mex>EcR RNAi*) and p=0.0001 (*mex*>w^{1118} vs. *mex>EcR RNAi, Hnf4)*; (**d**) p<0.0001. ***p≤0.001, ****p≤0.0001. Source data are provided as a Source Data file.

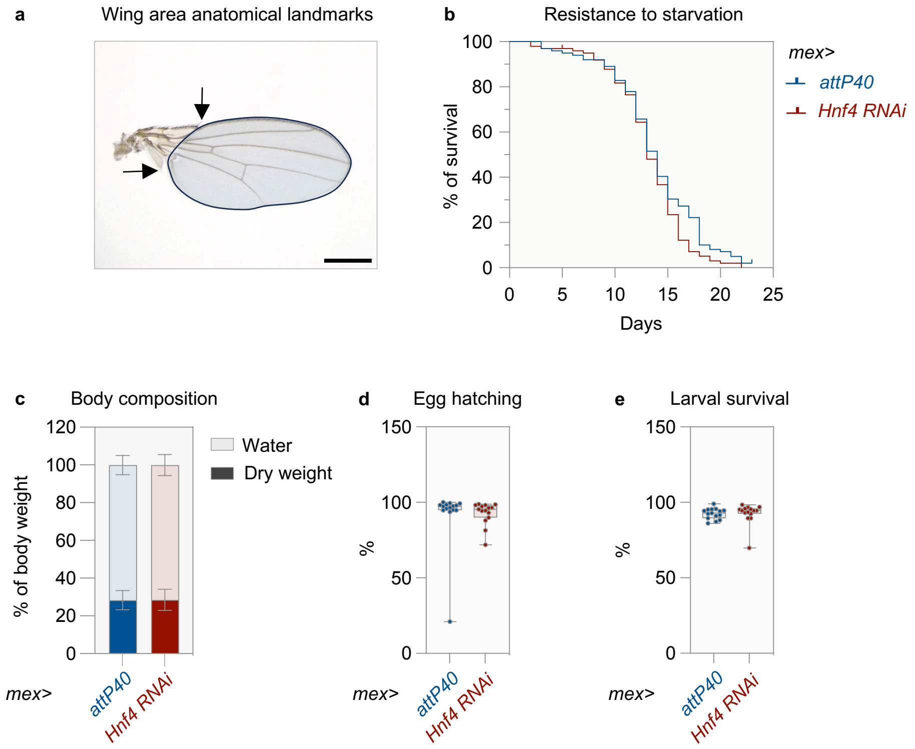
a
Wing area anatomical landmarks
b
Resistance to starvation
% of survival
Days
mex>
attP40
Hnf4 RNAi
c
Body composition
% of body weight
Water
Dry weight
mex>
attP40
Hnf4 RNAi
d
Egg hatching
%
mex>
attP40
Hnf4 RNAi
e
Larval survival
%
mex>
attP40
Hnf4 RNAi

Supplementary Figure 7. Chronic *Hnf4* silencing does not affect adult starvation resistance and fertility

(**a**) Anatomical landmarks for the measurement of wing area, related to (Fig. 7d). The arrows indicate the landmarks and the shaded area the portion of wing blade that was measured. Scale bar = 500 µm. (**b-e**) *Hnf4* was silenced in ECs by driving the expression of a *UAS-Hnf4 RNAi* with the *mex-GAL4* driver (*mex>Hnf4 RNAi*). Controls are the progenies of *mex-GAL4* females and males carrying the *attP40* site (*mex>attP40*). (**b**) Survival to wet starvation was scored daily in newly eclosed adult males. Median lifespan: *mex>attP40:* 14 days, *mex>Hnf4 RNAi:* 13 days. Values are plotted normalized to the total number of animals (n=100). One representative experiment out of three is shown. Censored animals are indicated by a tick mark on the Kaplan-Meier curves, see methods for details. (**c**) Water content was measured in newly eclosed adult males. n=15 biological replicates, each containing several animals, from three independent experiments (see the methods for details about the contents of the biological replicates used in these assays). (**d**) Hatching rate (48 hours after egg laying) and (**e**) survival until pupariation (10 days after egg laying) were scored for the progenies of control females and females with *Hnf4* silencing in ECs. (**d-e**) n=15 biological replicates (each vial initially seeded with embryos laid by five females considered as one biological replicate) from three independent experiments. The dots represent individual biological replicates, boxplots extend from the 25th to 75th percentile, whiskers extend from minimum to maximum, median is depicted as a line. (**b-e**) No statistically significant differences were found (**b, d and e**) between genotypes ((**b**) Mantel-Cox test, (**d and e**) Mann-Whitney test), or **(c)** between genotypes for water and dry weight (2-way ANOVA with Šidak's multiple comparisons). Exact p-values are indicated in the Source data file.

Supplementary Table 1. *Drosophila* lines

Line name	Provider	Reference
Drosophila melanogaster: attP40<br>*y[1] v[1]; P{y[+t7.7] = CaryP}Msp300[attP40]*	BDSC	RRID:BDSC_36304
Drosophila melanogaster: UAS-EcR^{DN}<br>w^{1118}*; P{w[+mC]=UAS-EcR.B1-DeltaC655.F645A}TP1*	Cherbas et al. (2003)[1]	RRID:BDSC_6869
Drosophila melanogaster: EcR-GFP<br>w^{1118}*; PBac{y[+mDint2] w[+mC]=EcR-EGFP.S}VK00033*	BDSC	RRID:BDSC_59040
Drosophila melanogaster: UAS-EcR RNA_i<br>w^{1118}*; P{GD1428}v37059*	VDRC	RRID:SCR_013805<br>Cat#37059
Drosophila melanogaster: UAS-Hnf4 RNA_i<br>*y[1] sc[*] v[1] sev[21]; P{y[+t7.7]*<br>*v[+t1.8]=TRiP.HMC05862}attP40*	BDSC	RRID:BDSC_64988
Drosophila melanogaster: UAS-Hnf4<br>w^{1118}*; UAS-Hnf4*	Palanker et al. (2009)[2]	N/A
Drosophila melanogaster: mex-GAL4<br>w^{1118}*; P{w[+mC]=mex1-GAL4.2.1}10-8*	BDSC	RRID:BDSC_ 91368
Drosophila melanogaster: UAS-Nplp2 RNA_i<br>w^{1118}*; P{GD4710}v15305*	VDRC	RRID:SCR_013805<br>Cat#15305
Drosophila melanogaster: tub^{ts}*>*<br>*w;P{tubP-GAL80ts};tub-GAL4/TM6B,tb*	Carl Thummel	N/A
Drosophila melanogaster: yw	Carl Thummel	N/A
Drosophila melanogaster: w^{1118}	Carl Thummel	N/A

Supplementary Table 2. Oligonucleotides for RT-qPCR

Gene	Forward sequence (5'-3')	Reverse sequence (5'-3')
Acox1	AATGGAGAGAAGCTGGTGCC	AATTCGGGTTTTGTTGGCGG
Agpat1	TCCGCAATCTGCTATGGGCG	AGCACATCCAGCGAGCTTT
Agpat2	TGCGTGGTGGCTTTAATCCT	AGCACCATGATGGAAAAAGCG
bgm	TGGACAAGATTCACGCCATTC	CGACCACCTGTAGTAGCCATC
bmm	ACGCACAGCAGCGACATGTAT	CTTTTCGCTTTGCTACGAG CC
br	TTGGCAGTGGCAGCAACAACAACA	GTGGTGCTTGATCGTACTGAAGTC
CG11162	CGACTATCACCATGCCAAGTTC	AGGAGCCTTTTGTTCTGCGG
CG31091	CAATGGTCCCGACACGATCC	CGGAAGAGGCTGATAATGTAGC
CG33120	GCAAACGAACGAGTCTGGGA	CCAAAGTGAGCACACAGGAAT
CG6271	TTTGGCAATCTTTGCTCTAGCCGC	ATCCATGTCCACCCACTCAAAGGA
CG6277	TTGCCGAACAGTGGATGGAAG	AAAGGTAGAACTTTACGGGAACG
CG6295	GAGGTTCGTGTGAATGGTGAG	CGGTTCTTGGTCTCCAAATAGG
Dgat2	GGCCATTAGAACGGGCTCATC	CTCCGGTGAGCTTCTTGAC
EcR	ACTCCAGCCACAGATTCAACCACA	CATGTATTCGCTGCTCGTACTGAC
Eo	CAGCAGACGCTACTTCGCTA	TGTATCGCATTCGTGGAGGG
Fatp3	GTCTATGGCGTGCAGATCCC	CAGCACCACGGACAGGTAAT
Gba1a	GGTCCCACGATCCGTAACTC	GTCGAGTGCGGTTCATCATT
Hacl	CAGTACGCAAGAAAACGCGG	GACATATTCCACGCCCTGTTG
Hnf4	TGGACAAGATTCACGCCATTC	CGCAAATCTGCAAGTGTACTGAT
Lip3	ATTGCGGTGAGCGCATTGA	TCAGGATGTAGTTGTCACTGGT
LpR1	TGCAACCAGACCTGTCGTG	TCGTGGTCGCACTTCCATC
LpR2	GAAATAGCCTTGCATGTGATTGC	GTGGTAGACGGGATTCTCGAA
Lsd-1	CAGCGCATACCACTGGTCTAT	GCATTACCGATTTGCTTGACAG
mag	CTCGCCGAGAAGTCGGACTA	CTCGTGAGTCTCCGTGGGATA
Mfe2	GTCCTGCGAGGATAATGGTAGC	ACGTACTCAATGGTCACGGG
mino	TGCGTGGTGGCTTTAATCCT	AGCACCATGATGGAAAAAGCG
Npc2a	GGCGGAGTGCATCCTCAAG	CGTGGACGACTGTCTTCACC
Npc2c	CAACTATCCTCAGCCACTGATG	CCTCGGTTCCCTTCCACAAAT
Npc2f	GATTGCGGTTCTTTGTACCAGG	GGTGAACTTGCCCGTCTTC
pdgy	CGGGACTGACGGTGACCA	CGGATACCGTTCCCACCA
Pepck2	CCCATCCCAACTCCAGGTTC	CAAGGGAACTCCACTGGGAC
rp49	GACGCTTCAAGGGACAGTATCTG	AACGCGGTTCTGCATGA
Seipin	CCGTGGTGCTCATCATCTGG	GTCTCCAGGCAGGTTTTGAACT
shd	TCGCTTAATGCAGGGACTGT	CCGAAGGGCACCACAATACT
tbp	AAGCTCGGTTTCCCTGCAA	TCCAGTGAGCACCACCTTTC
yip2	AAATGTGATCGCGTCCTCCTC	GCCGGTAAGGGCTATCTTGG

Supplementary References

1. Cherbas, L., Hu, X., Zhimulev, I., Belyaeva, E. & Cherbas, P. EcR isoforms in *Drosophila*: testing tissue-specific requirements by targeted blockade and rescue. *Development* **130**, 271–284 (2003).
2. Palanker, L., Tennessen, J. M., Lam, G. & Thummel, C. S. *Drosophila* HNF4 regulates lipid mobilization and beta-oxidation. *Cell Metab* **9**, 228–39 (2009).
