# Supplementary material for: Transient remodeling of gut metabolism supports juvenile growth and adult fitness in Drosophila
Source: Nat Commun. 2026 Apr 13;17:3458. doi: 10.1038/s41467-026-71776-3 (PMC13077097; doi:10.1038/s41467-026-71776-3)
Supplement: Supplementary file 2 — Description of Additional Supplementary Files [file 41467_2026_71776_MOESM2_ESM.pdf]

Description of Additional Supplementary Files

File Name: Supplementary Data 1

Description: mRNA-seq analysis of developing *w*^{1118} larval midguts and gene clusters.

File Name: Supplementary Data 2

Description: Gene ontology term enrichment analysis (Biological Processes) on gene clusters shown in (Fig. 2A).

File Name: Supplementary Data 3

Description: List of *Drosophila* genes encoding putative digestive enzymes.

File Name: Supplementary Data 4

Description: Transcription Factor binding site analysis on the list of genes belonging to the different clusters identified in (Fig. 2a).

File Name: Supplementary Data 5

Description: mRNA-seq analysis of developing larval midguts with *Hnf4* suppression in ECs.

File Name: Supplementary Data 6

Description: Peak locations of ecdysone-related transcription factors within 5 kb of the transcription start site of the *Hnf4* locus.
