# Supplementary material for: Transient remodeling of gut metabolism supports juvenile growth and adult fitness in Drosophila
Source: Nat Commun. 2026 Apr 13;17:3458. doi: 10.1038/s41467-026-71776-3 (PMC13077097; doi:10.1038/s41467-026-71776-3)
Supplement: Supplementary file 9 — Reporting Summary [file 41467_2026_71776_MOESM9_ESM.pdf]

nature portfolio

Gilles Storelli

Corresponding author(s): gilles.storelli@cos.uni-heidelberg.de

Last updated by author(s): Mar 6, 2026

Reporting Summary

Nature Portfolio wishes to improve the reproducibility of the work that we publish. This form provides structure for consistency and transparency in reporting. For further information on Nature Portfolio policies, see our Editorial Policies and the Editorial Policy Checklist.

Statistics

For all statistical analyses, confirm that the following items are present in the figure legend, table legend, main text, or Methods section.

n/a	Confirmed	
☐	☒	The exact sample size (*n*) for each experimental group/condition, given as a discrete number and unit of measurement
☐	☒	A statement on whether measurements were taken from distinct samples or whether the same sample was measured repeatedly
☐	☒	The statistical test(s) used AND whether they are one- or two-sided<br>*Only common tests should be described solely by name; describe more complex techniques in the Methods section.*
☒	☐	A description of all covariates tested
☐	☒	A description of any assumptions or corrections, such as tests of normality and adjustment for multiple comparisons
☐	☒	A full description of the statistical parameters including central tendency (e.g. means) or other basic estimates (e.g. regression coefficient) AND variation (e.g. standard deviation) or associated estimates of uncertainty (e.g. confidence intervals)
☐	☒	For null hypothesis testing, the test statistic (e.g. *F*, *t*, *r*) with confidence intervals, effect sizes, degrees of freedom and *P* value noted<br>*Give P values as exact values whenever suitable.*
☒	☐	For Bayesian analysis, information on the choice of priors and Markov chain Monte Carlo settings
☒	☐	For hierarchical and complex designs, identification of the appropriate level for tests and full reporting of outcomes
☒	☐	Estimates of effect sizes (e.g. Cohen's *d*, Pearson's *r*), indicating how they were calculated

Our web collection on statistics for biologists contains articles on many of the points above.

Software and code

Policy information about availability of computer code

Data collection	SkanIt (v5.0) Thermo Fisher Scientific https://www.thermofisher.com<br>Zen 2009 Zeiss https://www.zeiss.com/microscopy/en/products/software/light-microscopy-software.html<br>cellSens Standard Software (Olympus, v1.11)

Data analysis	Cytoscape (v.3.10.1) RRID:SCR_003032<br>Fiji (v.1.54p) RRID:SCR_002285<br>Fiji macro: InterEdgeDistance (v2.0) https://sourceforge.net/projects/interedgedistance/<br>Graphpad Prism (v10) Graphpad Software RRID:SCR_002798<br>iDEP (v0.96) http://bioinformatics.sdstate.edu/idep96/<br>iRegulon (v1.3) Jhttps://apps.cytoscape.org/apps/iregulon<br>MultiQC (v1.11) RRID:SCR_014982<br>FastQC Babraham Bioinformatics RRID:SCR_014583<br>Picard Broad Institute RRID:SCR_006525<br>Preseq RRID:SCR_018664<br>STAR (v2.7.10a) RRID:SCR_004463<br>Salmon (v1.5.2) RRID:SCR_017036<br>DESeq2 RRID:SCR_015687<br>TF2TG online portal (https://www.flyrnai.org/tools/tf2tg/web/)

For manuscripts utilizing custom algorithms or software that are central to the research but not yet described in published literature, software must be made available to editors and reviewers. We strongly encourage code deposition in a community repository (e.g. GitHub). See the Nature Portfolio guidelines for submitting code & software for further information.

Data

Policy information about availability of data

All manuscripts must include a data availability statement. This statement should provide the following information, where applicable:

- Accession codes, unique identifiers, or web links for publicly available datasets
- A description of any restrictions on data availability
- For clinical datasets or third party data, please ensure that the statement adheres to our policy

mRNA-seq data generated in this study have been deposited in the Gene Expression Omnibus (GEO) database under the following accession codes: GSE283125 and GSE315710.
https://www.ncbi.nlm.nih.gov/geo/query/acc.cgi?acc=GSE283125
https://www.ncbi.nlm.nih.gov/geo/query/acc.cgi?acc=GSE315710
Source Data are provided with this paper.

Research involving human participants, their data, or biological material

Policy information about studies with human participants or human data. See also policy information about sex, gender (identity/presentation), and sexual orientation and race, ethnicity and racism.

Reporting on sex and gender	Not applicable
Reporting on race, ethnicity, or other socially relevant groupings	Not applicable
Population characteristics	Not applicable
Recruitment	Not applicable
Ethics oversight	Not applicable

Note that full information on the approval of the study protocol must also be provided in the manuscript.

Field-specific reporting

Please select the one below that is the best fit for your research. If you are not sure, read the appropriate sections before making your selection.

[x] Life sciences [ ] Behavioural & social sciences [ ] Ecological, evolutionary & environmental sciences

For a reference copy of the document with all sections, see nature.com/documents/nr-reporting-summary-flat.pdf

Life sciences study design

All studies must disclose on these points even when the disclosure is negative.

Sample size	No statistical methods were used to predetermine sample size. Sample sizes were chosen based on experimental standards in the field.
Data exclusions	No data or subjects were excluded from our analyses.
Replication	Experiments were repeated independently three times, unless otherwise stated in the Figure legends. There were no issues with replication.
Randomization	Animals were allocated to study groups based on their genotype and age. Within a study group, animals were collected from independent

vials to make up biological replicates.

Blinding

Blinding could not be used during of our study because the same researcher (CL) was responsible for collecting animals, generating the samples and analysing them.

Reporting for specific materials, systems and methods

We require information from authors about some types of materials, experimental systems and methods used in many studies. Here, indicate whether each material, system or method listed is relevant to your study. If you are not sure if a list item applies to your research, read the appropriate section before selecting a response.

Materials & experimental systems

n/a	Involved in the study
☐	☒ Antibodies
☒	☐ Eukaryotic cell lines
☒	☐ Palaeontology and archaeology
☐	☒ Animals and other organisms
☒	☐ Clinical data
☒	☐ Dual use research of concern
☒	☐ Plants

Methods

n/a	Involved in the study
☒	☐ ChIP-seq
☒	☐ Flow cytometry
☒	☐ MRI-based neuroimaging

Antibodies

Antibodies used

Anti-HNF4 Guinea Pig antibody, Affinity-purified. Palanker et al. (2009)
1/150 (v/v) in PBS with 0.5% Triton-X100 (Merck, Cat#1,086,031,000)

Anti-GFP (5G4) Mouse mAb Cell Signaling Technology RRID:AB_3101977
1/500 (v/v) in PBS with 0.5% Triton-X100 (Merck, Cat#1,086,031,000)

Cy™3 AffiniPure® Donkey Anti-Guinea Pig IgG (H+L) Jackson ImmunoResearch RRID:AB_2340460
1/500 (v/v) in PBS with 0.5% Triton-X100 (Merck, Cat#1,086,031,000)

Cy™3 AffiniPure® Donkey Anti-Mouse IgG (H+L) Jackson ImmunoResearch RRID:AB_2315777
1/500 (v/v) in PBS with 0.5% Triton-X100 (Merck, Cat#1,086,031,000)

Validation

Anti-HNF4 Guinea Pig antibody, Affinity-purified
Validated in Palanker et al. (2009)

GFP (5G4) Mouse Monoclonal Antibody
Detects native GFP and YFP-tagged proteins exogenously expressed in cells, but does not detect denatured GFP.

Cy™3 AffiniPure® Donkey Anti-Guinea Pig IgG (H+L) Jackson ImmunoResearch RRID:AB_2340460
Based on immunoelectrophoresis and/or ELISA, the antibody reacts with whole molecule guinea pig IgG. It also reacts with the light chains of other guinea pig immunoglobulins. No antibody was detected against non-immunoglobulin serum proteins. The antibody has been tested by ELISA and/or solid-phase adsorbed to ensure minimal cross-reaction with bovine, chicken, goat, syrian hamster, horse, human, mouse, rabbit, rat and sheep serum proteins, but it may cross-react with immunoglobulins from other species.

Cy™3 AffiniPure® Donkey Anti-Mouse IgG (H+L) Jackson ImmunoResearch RRID:AB_2315777
Based on immunoelectrophoresis and/or ELISA, the antibody reacts with whole molecule mouse IgG. It also reacts with the light chains of other mouse immunoglobulins. No antibody was detected against non-immunoglobulin serum proteins. The antibody has been tested by ELISA and/or solid-phase adsorbed to ensure minimal cross-reaction with bovine, chicken, goat, guinea pig, syrian hamster, horse, human, rabbit, rat and sheep serum proteins, but it may cross-react with immunoglobulins from other species.

Animals and other research organisms

Policy information about studies involving animals; ARRIVE guidelines recommended for reporting animal research, and Sex and Gender in Research

Laboratory animals

Drosophila melanogaster: attP40
y[1] v[1]; P{y[+t7.7] = CaryP}Msp300[attP40] BDSC RRID:BDSC_36304

Drosophila melanogaster: UAS-EcRDN
w1118; P{w[+mC]=UAS-EcR.B1-DeltaC655.F645A}TP1 (Cherbas et al. (2003) RRID:BDSC_6869)

Drosophila melanogaster: EcR-GFP
w1118; PBac{y[+mDint2] w[+mC]=EcR-EGFP.S}VK00033 BDSC RRID:BDSC_59040

Drosophila melanogaster: UAS-EcR RNAi
w1118; P{GD1428}v37059 VDRC RRID:SCR_013805 Cat#37059

Drosophila melanogaster: UAS-Hnf4 RNAi
y[1] sc[*] v[1] sev[21]; P{y[+t7.7] v[+t1.8]=TRiP.HMC05862}attP40 BDSC RRID:BDSC_64988

Drosophila melanogaster: UAS-Hnf4
w1118; UAS-Hnf4 Palanker et al. (2009)

Drosophila melanogaster: mex-GAL4
w1118; P{w[+mC]=mex1-GAL4.2.1}10-8 BDSC RRID:BDSC_ 91368

Drosophila melanogaster: UAS-Nplp2 RNAi
w1118; P{GD4710}v15305 VDRC RRID:SCR_013805 Cat#15305

Drosophila melanogaster: tubts-GAL4
w;P{tubP-GAL80ts};tub-GAL4/TM6B,tb

Drosophila melanogaster: yw

Drosophila melanogaster: w1118

Wild animals

The study did not involve wild animals

Reporting on sex

The study involved sex-specific investigations in adult Drosophila. The sex of adult Drosophila is indicated in the Figure legends, main text and methods wherever appropriate.
Studies were also performed in larvae, at a stage when sex cannot be determined. Therefore, studies were performed on groups of larvae with undetermined sex. All details are indicated in the legends, main text and methods.

Field-collected samples

The study did not involve samples collected from the field

Ethics oversight

The study did not require ethical approval

Note that full information on the approval of the study protocol must also be provided in the manuscript.

Plants

Seed stocks

Not applicable

Novel plant genotypes

Not applicable

Authentication

Not applicable
